# Numerical Simulation and Deformation Prediction of Deep Pit Based on PSO-BP Neural Network Inversion of Soil Parameters

**DOI:** 10.3390/s24102959

**Published:** 2024-05-07

**Authors:** Qingwang Li, Feng Cheng, Xinran Zhang

**Affiliations:** School of Architecture and Transportation Engineering, Guilin University of Electronic Science and Technology, Guilin 541004, China; liqingwang99@163.com (Q.L.); zhangxr@mails.guet.edu.cn (X.Z.)

**Keywords:** deep pit, neural networks, Particle Swarm Optimization, soil layer parameter inversion, numerical simulation

## Abstract

The finite element numerical simulation results of deep pit deformation are greatly influenced by soil layer parameters, which are crucial in determining the accuracy of deformation prediction results. This study employs the orthogonal experimental design to determine the combinations of various soil layer parameters in deep pits. Displacement values at specific measurement points were calculated using PLAXIS 3D under these varying parameter combinations to generate training samples. The nonlinear mapping ability of the Back Propagation (BP) neural network and Particle Swarm Optimization (PSO) were used for sample global optimization. Combining these with actual onsite measurements, we inversely calculate soil layer parameter values to update the input parameters for PLAXIS 3D. This allows us to conduct dynamic deformation prediction studies throughout the entire excavation process of deep pits. The results indicate that the use of the PSO-BP neural network for inverting soil layer parameters effectively enhances the convergence speed of the BP neural network model and avoids the issue of easily falling into local optimal solutions. The use of PLAXIS 3D to simulate the excavation process of the pit accurately reflects the dynamic changes in the displacement of the retaining structure, and the numerical simulation results show good agreement with the measured values. By updating the model parameters in real-time and calculating the pile displacement under different working conditions, the absolute errors between the measured and simulated values of pile top vertical displacement and pile body maximum horizontal displacement can be effectively reduced. This suggests that inverting soil layer parameters using measured values from working conditions is a feasible method for dynamically predicting the excavation process of the pit. The research results have some reference value for the selection of soil layer parameters in similar areas.

## 1. Introduction

Numerical simulation was first applied to model one-dimensional unstable radial and linear flow [1,2]. With the enhancement of computing capabilities, Clough et al. [3] first applied finite element software to pit engineering, proposing the parameterized finite element method and concluding that optimizing support structure parameters can effectively reduce the horizontal displacement of retaining structures. Numerical simulation effectively models the interaction between soil and support structures during the excavation of pits [4,5,6,7,8] and offers excellent three-dimensional (3D) visualization effects. Neural networks in pit deformation monitoring, with field monitoring data as the learning object, are relatively simple to model, and the prediction results are more refined on the time scale [9,10,11,12,13]. Currently, most scholars studying deformation predictions of deep pits caused by excavation solely use either numerical simulation or machine learning methods. Single analysis methods have certain limitations in addressing the complex geological environments and engineering characteristics of deep pit projects.

The method of parametric inverse analysis is widely used in engineering and slope engineering under complex geological conditions [14,15,16,17]. Zhang et al. used different theoretical methods to analyze the distribution law of soil pressure on the side of the diaphragm wall and verified the applicability of the theoretical methods, which was in good agreement with the measured values [18,19], which provides a certain theoretical basis for this paper to use finite element numerical simulation to carry out parameter sensitivity analysis. Zhang et al. [20] employed a design method for resisting the overturning of rigid retaining walls in unsaturated soil excavations and verified the applicability of the adopted approach. Ramakrishna Annapareddy et al. [21] developed a novel method reliant on suction stress for calculating horizontal and vertical seismic acceleration profiles within backfilled soil, and they validated the feasibility of this method.

In recent years, to provide crucial technical support for the design and construction safety of pits, scholars have combined finite element numerical simulation with neural networks. By inverting soil layer parameters through neural network models, they have optimized input parameters for finite element software, achieving significant research outcomes [22,23,24,25,26,27,28,29,30]. Ling et al. [22] proposed an intelligent displacement back-analysis method based on improved PSO and BP neural networks. By integrating measured displacement data from surrounding rocks at the construction site, they successfully inverted the mechanical parameters of the tunnel surrounding rocks, demonstrating the feasibility of this method. Kwang et al. [24] used actual displacement monitoring data and combined finite element and neural network methods to invert geotechnical parameters, obtaining optimal parameters and confirming the reliability of this method. Li et al. [25] utilized the Mind Evolutionary Computation (MEC) algorithm to optimize initial weights and thresholds of BP neural networks, combined with finite element numerical simulation, proposed a soil layer parameter inversion analysis method based on horizontal displacement of pits and verified the stability and accuracy of the results. Wang et al. [26], based on actual data from a subway tunnel, used a BP neural network to invert the physical and mechanical parameters of the soil. The calculated results were input into the MIDAS GTS NX software for forward numerical simulation, predicting deformations in subway tunnel structures due to excavation. Zou et al. [27] used the finite difference numerical simulation software to conduct numerical simulation calculations for a deep pit of a station, built a training set based on the numerical simulation results, used the BP neural network to invert the formation parameters of the deep pit of the station, and applied the inversion parameters to the construction to verify the rationality of the method. Ma et al. [28] designed 64 representative soil parameter combinations through orthogonal experiments, obtained the displacement value of each parameter combination through finite element numerical calculation, used the actual monitoring data to invert the soil parameter of dynamic deformation of the pit, obtained the deformation data of the next excavation stage, and verified the accuracy and feasibility of this method. Zhao et al. [29] used PLAXIS finite element software to simulate and analyze the deformation characteristics of deep pit excavation and, combined with the Sparrow Search Algorithm BP(SSA-BP) neural network, proposed the parameter back analysis method of the HSS model in the Dalian area. The results showed that, under the premise of reasonable parameter selection. The finite element analysis results of the Hardening soil-Small (HSS) model considering the characteristics of Soil small strain were highly consistent with the actual deformation laws of the pit, which proved the rationality of the parameter reverse analysis.

Analysis of current research, both domestically and internationally, reveals that the accuracy of numerical simulations is greatly influenced by the input parameters of the soil layers, and the three-dimensional visualization effects of neural network predictions are suboptimal. At present, most scholars only invert specific soil layer parameters. However, during the excavation of deep pits, the spatial shape, excavation area, and depth of the pit are constantly changing. Therefore, it becomes particularly important to develop multifaceted coupled numerical simulation techniques that closely align with the practical realities of engineering projects.

This study is based on a deep pit project and conducts a sensitivity analysis of soil layer parameters. Using orthogonal testing, different parameter combinations are derived. These combinations are then utilized in PLAXIS 3D to compute displacement values at various sensor points, thus generating training samples. By leveraging the powerful nonlinear mapping capabilities of neural networks and the global optimization features of PSO and integrating actual onsite measurement values, the study successfully inverts the parameters of multiple soil layers. The inverted parameters are subsequently used as input parameters in PLAXIS 3D, enabling dynamic prediction throughout the entire excavation process of the pit.

## 2. Fundamental Principles

### 2.1. Displacement Calculation in PLAXIS 3D

PLAXIS 3D V20 Update 1 is a three-dimensional geological modeling and numerical analysis software based on the finite element method. In displacement calculations, the model is discretized into a finite number of elements, and a system of linear equations is solved to determine the displacement and stress state of each element. Based on the solutions obtained, the deformations of the soil and the retaining structures during the excavation process can be computed.

### 2.2. Neural Networks

#### 2.2.1. BP Neural Networks

**Figure 1:**
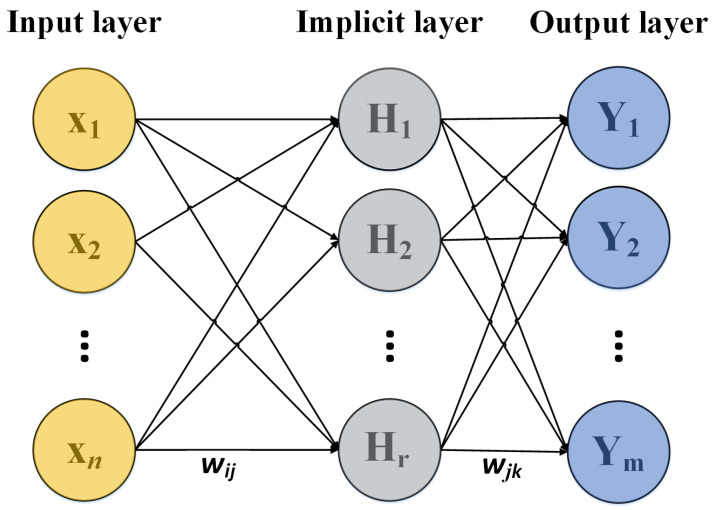
Basic structure of BP neural network.

The BP neural network is a multi-layer feedforward neural network trained using the error backpropagation algorithm. It primarily consists of an input layer, hidden layers, and an output layer. The process of backpropagation in a BP neural network involves continuously adjusting the weights and thresholds based on the error values. The basic structure of the BP neural network is illustrated in Figure 1.

In Figure 1, the BP neural network’s input values are denoted as $X_{1},X_{2},\ldots ,X_{n}$, and the output values as $Y_{1},Y_{2},\ldots ,Y_{n}$, the weights of the BP neural network are represented by $w_{ij}$ and $w_{jk}$, while n represents the number of nodes in the input layer and m the number of nodes in the output layer. The specific training steps of the BP neural network are as follows:

Step 1: Network Initialization; assume the initial thresholds of the network are a and b;

Step 2: Hidden Layer Output Calculation; the formula is as follows:

(1) $$H_{j}=f(\sum_{i=1}^{n}w_{ij}x_{i}-a_{j}),j=1,2,\ldots ,l$$

In the formula: $w_{ij}$ and a respectively represent the weights and thresholds between the input layer and the hidden layer of the BP neural network, l represents the number of nodes in the hidden layer, and f is the activation function of the hidden layer.

Step 3: Output Layer Calculation: the formula for calculating the neural network’s predictive output is as follows:

(2) $$H_{k}=\sum_{j=1}^{l}H_{j}w_{jk}-b_{k},k=1,2,\ldots ,m$$

In the formula, $w_{jk}$ and b represent the connection weights and thresholds of the neural network, respectively.

Step 4: Error Calculation: the error e is calculated based on the predicted output H and the desired output Y of the neural network. The calculation formula is as follows:

(3) $$e_{k}=Y_{k}-H_{k},k=1,2,\ldots ,m$$

Step 5: Updating the Weights of the Input and Hidden Layers. Update the network connection weights $w_{ij}$ and $w_{jk}$ using the predicted error e from the BP neural network. The calculation formula is as follows:

(4) $$w_{ij}=w_{ij}+\eta H_{j}(1-H_{j})x(i)\sum_{k=1}^{m}w_{jk}e_{k},i=1,2,\ldots ,m,j=1,2,\ldots ,m$$

In the formula: η is the learning rate.

Step 6: Judge whether the algorithm is finished or not; otherwise, return to Step 2 until the end.

#### 2.2.2. Particle Swarm Optimization (PSO)

**Figure 2:**
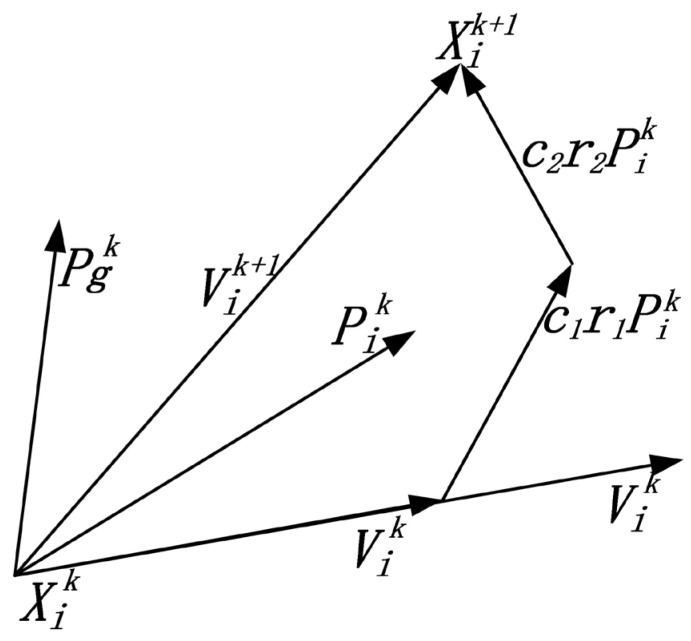
Schematic of particle position update.

The PSO is primarily inspired by observations of bird predation behavior. The principle of the algorithm is as follows:

Suppose there are m particles in D-dimensional space, with the position of the particle i(i=1,2,…,m) given by $X_{i}(X_{i1},X_{i2},\ldots ,X_{iD})$ and velocity by $V_{i}(V_{i1},V_{i2},\ldots ,V_{iD})$. The fitness value is calculated by inserting $X_{i}$ into the objective function to assess the quality of the particle’s position. The optimal position during a single particle’s flight is denoted as $P_{i}(P_{i1},P_{i2},\ldots ,P_{iD})$, comparing the flight experiences of all particles in the population, and the best group position is determined as $P_{g}(P_{g1},P_{g2},\ldots ,P_{gD})$, g=1,2,…,m. Each particle updates its velocity and position according to Formulas (5) and (6). The schematic diagram of particle position updates is shown in Figure 2.

(5) $$V_{i}^{k+1}=\omega V_{i}^{k}+c_{1}r_{1}(P_{i}^{k}-X_{i}^{k})+c_{2}r_{2}(P_{g}^{k}-X_{i}^{k})$$

(6) $$X_{i}^{k+1}=X_{i}^{k}+V_{i}^{k+1}$$

In the formula, ω is the inertia weight factor, $c_{1},c_{2}$ is the learning factor, r is a random number in the interval [0,1], and k is the number of iterations.

Formula (5) indicates that the velocity update for particle i is primarily determined by three factors: the velocity of particle i at the previous moment, labeled as $V_{i}^{k}$; the distance between the current position of particle i and its optimal position, denoted as $P_{i}^{k}-X_{i}^{k}$; and the distance between the current position of particle i and the overall best position, marked as $P_{g}^{k}-X_{i}^{k}$.

#### 2.2.3. PSO-BP Neural Networks

**Figure 3:**
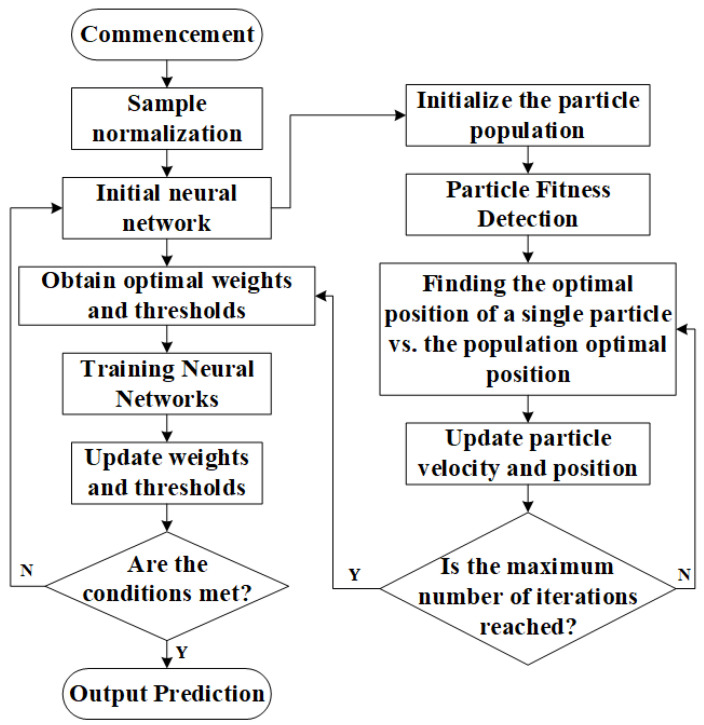
PSO-BP Neural Network Process.

PSO is a straightforward and easy-to-implement method for parameter tuning, primarily focused on searching for the optimal initial weights and thresholds. To address issues such as significant errors and the tendency of traditional BP neural networks to converge to local minima, PSO is employed to optimize BP neural networks. The implementation process of the algorithm is illustrated in Figure 3.

### 2.3. Inversion of Soil Layer Parameters

Utilizing the powerful nonlinear mapping capabilities of neural networks along with the global optimization features of PSO enables accurate and rapid determination of soil layer parameter values. The steps of the parameter inversion method adopted in this study are as follows:

Step 1: Sample Collection

Through orthogonal experimentation, different parameter value combinations are obtained. PLAXIS 3D is used to calculate displacement values at sensor points under these different parameter combinations, generating displacement data for each parameter group, which serve as training samples for the neural network.

Step 2: Construction of Nonlinear Function Relationships

Machine learning algorithms are written by using MATLAB V9.10 software. To accelerate the convergence of the neural network, input data are normalized, and output data are reverse normalized. The number of nodes in the hidden layer is determined, and a transfer function suitable for each set of parameters and their corresponding displacement values is defined.

Step 3: Sample Output and Testing

Displacement values measured at specific sensor points under a certain working condition are used as input samples. The output parameter combinations, which are the inverted soil layer parameters, are then used to update the input parameters in PLAXIS 3D and perform calculations to predict deformations for the next working condition.

## 3. Engineering Overview

### 3.1. Excavation Profile

The pit is irregular, with a total area of approximately 8000 m^2^. The excavation depth of the pit ranges from 10.95 m to 11.90 m, with a maximum length of 103.5 m and a maximum width of 80 m. Ground elevations around the site are set at −0.300 m (relative elevation). The top elevation of the basement floor slab in the second basement is −9.95 m., with a thickness of 0.8 m and a cushion layer thickness of 0.1 m. The bottom elevation of the basement floor cushion layer is −10.85 m. The thickness of the bearing platform at the edge of the pit is 1.2 m and 1.8 m, respectively, with the bottom elevation of the platform cushion layer ranging from −11.25 m to −11.85 m. The bottom elevation of the cushion layer of the partial edge sump floor is −12.20 m. The excavation depth of the elevator pits in the water collecting wells and other pits within the pit ranges from 0.85 m to 3.45 m. Based on the severity of the impact on the surrounding environment and underground structures due to the excavation depth, support structure damage, soil instability, or excessive deformation, the safety level of this pit is classified as Grade I.

### 3.2. Geological Conditions

**Figure 4:**
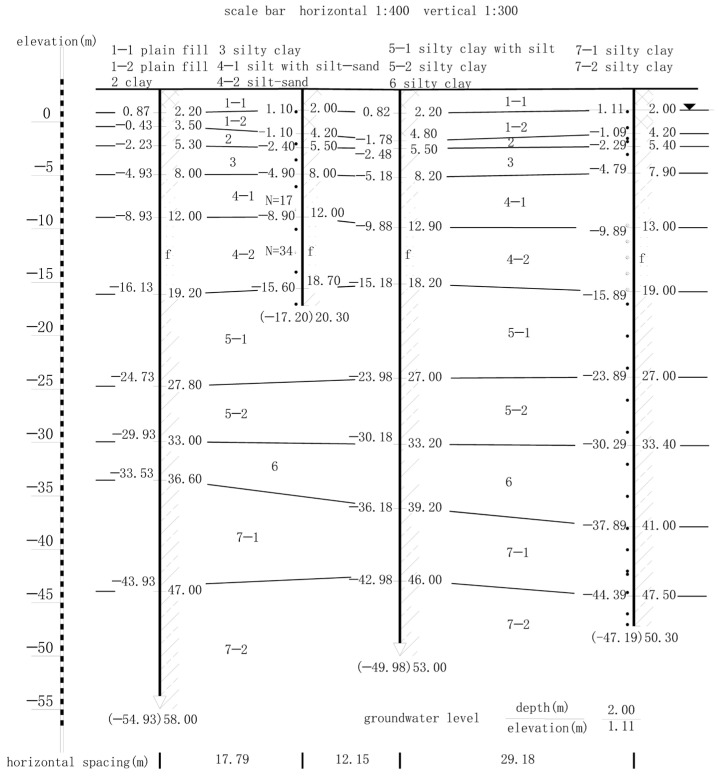
Geological Cross-Section.

The maximum depth of exploration is 90.75 m below the natural ground surface of the site, and the soils at the shallow depth, except for the soil fill, consist of the Quaternary Holocene to Middle to Late Pleistocene alluvial to lacustrine clastic sediments. The survey report shows that the soil layer around the pit from the top down is 1-1 plain fill, 1-2 plain fill, 3 clay, 4-1 silty clay, 4-2silt with silt-sand. The physical and mechanical parameters of each soil layer are listed in Table 1, and a typical geological profile is shown in Figure 4.

### 3.3. Hydrogeological Conditions

The surface water system in the area is well-developed, with a dense network of rivers and lakes. The fluctuations in river and lake water levels generally correspond to the inter-annual and intra-annual variations in precipitation, albeit with some lag. The groundwater level remains consistently higher than the surface water level, exhibiting the characteristic of unidirectional discharge into rivers and lakes. Shallow confined aquifers exist in the silt and fine sand layers, and their dynamics are influenced by factors such as atmospheric precipitation, topography, and surface water bodies, exhibiting characteristics of precipitation-induced fluctuations. Pre-excavation precipitation measures are undertaken, and the groundwater types relevant to this project mainly include phreatic water and micro-confined water. Refer to the typical geological section in Figure 4 for details.

### 3.4. Surrounding Environment and Protection Scheme

To the east of the pit lies an approximately 8.0 m wide municipal road, while at the northeast corner stands a 15-story existing building; the nearest distance from the proposed pit is 5.0 m. The southern and western sides of the site are bordered by municipal roads, and to the north, there are municipal roads and a market building.

The protection system for this pit involves the use of a bored pile/biting pile combined with two layers of reinforced concrete internal support. The piles are treated with shotcrete protection, supplemented by fully enclosed Cutter Soil Mixing cement-soil mixed curtain walls for waterproofing. The bored piles are constructed flush with the inner side of the excavation, with a clear spacing of 0.2 m between piles and a 0.2 m clear spacing between the cement-soil mixed curtain wall and the bored piles. The support system utilizes bracing, counter-bracing, and edge trusses, along with steel columns supplemented by bored piles as the vertical support structure for the horizontal support system. Drainage in the pit using pipe wells to evacuate dry precipitation and open ditch catchment wells to pump water. The bored piles and biting piles are constructed using underwater C30 concrete, while the crown beams, wale, support beams, and biting file guide walls are constructed with C30 concrete and the sprayed concrete surface layer with C20. Reinforcement bars are of HPB300 and HRB400 grades, and the cement is P.O42.5 ordinary Portland cement.

### 3.5. Pit Monitoring Design

**Figure 5:**
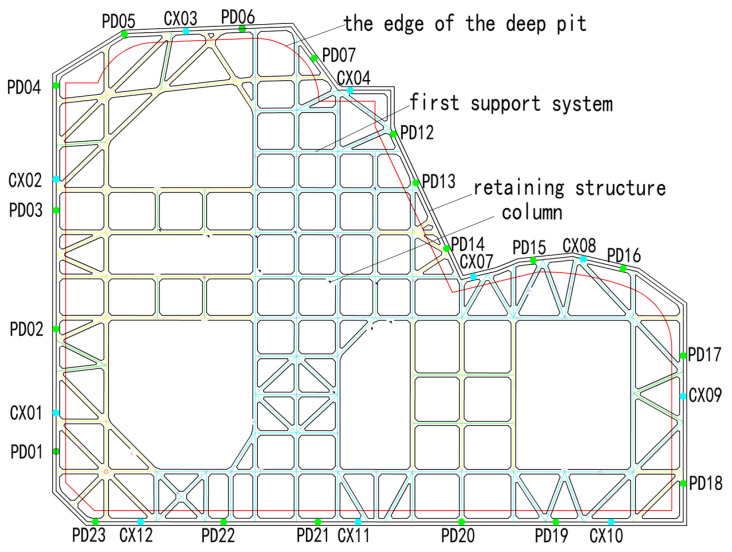
Pit Plan.

Monitoring focuses on the pit support structure, the bottom and surrounding soil of the pit, surrounding buildings, relevant pipelines and facilities, municipal structures, groundwater conditions, and important surrounding roads. Monitoring continues until the completion of the pit, with a frequency of once per day. This study primarily calculates the vertical displacements of pile tops (PD01–PD23) and the deep horizontal displacements of piles (CX01–CX12) to assess whether the accuracy of numerical simulation predictions improves after parameter inversion. Vertical displacement monitoring employs the leveling method using an electronic level; deep horizontal displacement monitoring of the support structure utilizes inclinometers, with the bottom of the inclinometer casing serving as the reference point for monitoring the deformation of the support structure. The layout of the pit plan and monitoring points is illustrated in Figure 5.

## 4. Numerical Simulation Results and Sensitivity Analysis of Parameters

### 4.1. Finite Element Model

The dimensions of the finite element model were determined according to specifications and reference to similar engineering experiences, considering the influence of hydrogeological conditions and in conjunction with engineering background materials. The model dimensions were set at 340 m × 280 m × 100 m. The establishment of the model incorporated the actual excavation process of the pit and the constitutive relationship of the soil adopted the small strain HSS model. Plate elements in PLAXIS 3D can withstand vertical loads and possess the ability to resist bending and elastic deformation. Therefore, plate elements were used to simulate the support of the row pile. The thickness of the plate element was calculated to be 0.7 m using the formula for stiffness equivalent conversion Equation (7). Interface elements were used to simulate the waterproof curtain and the interaction between the soil and the structure. The boundary of the pit was assumed to be impermeable. Internal support using steel support equivalent reinforced concrete internal support and embedded beam elements were used to simulate the vertical support structure of the horizontal support system.

(7) $$\frac{1}{12}(D+t)h^{3}=\frac{1}{12}\pi D^{4}$$

In the Equation, t represents the pile spacing, D represents the pile diameter, and h represents the equivalent thickness.

In the HSS constitutive model, the soil stiffness parameters, including the reference stiffness modulus $E_{50}^{ref}$, modulus of unloading and reloading $E_{ur}^{ref}$, and reference tangent modulus $E_{oed}^{ref}$, are derived from laboratory soil compression modulus tests. The relationship among these parameters [31] is referenced from Equation (8):

(8) $$E_{50}^{ref}\approx E_{ur}^{ref};E_{oed}^{ref}=3\sim 5E_{50}^{ref}$$

**Figure 6:**
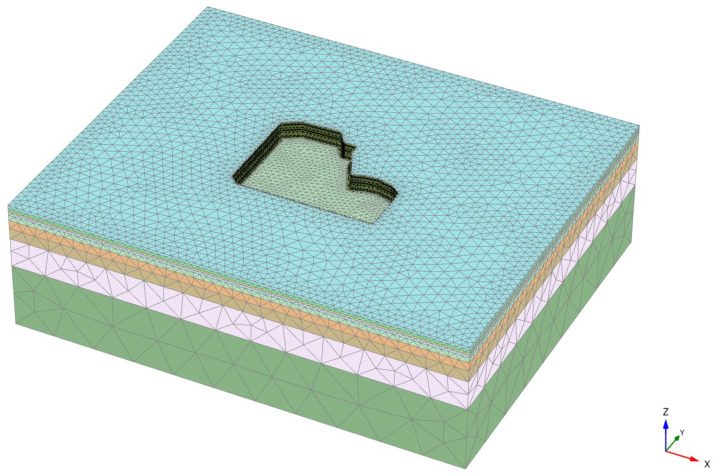
Finite element model of the pit.

**Figure 7:**
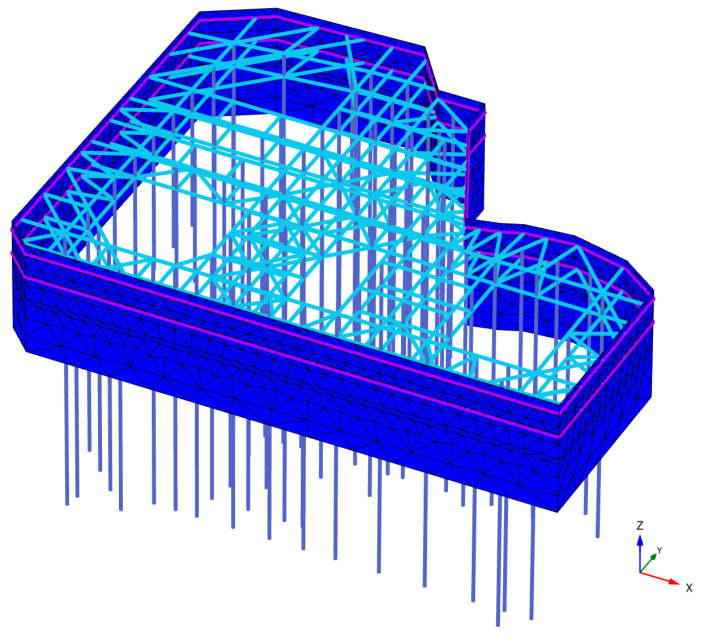
Support Structure Diagram.

The model constructed using PLAXIS 3D finite element software is depicted in Figure 6, while the support structure is illustrated in Figure 7. A total of 169,360 elements and 257,191 nodes were generated. The model’s computational boundary conditions are based on the excavation of the pit. It is assumed that dewatering is completed during excavation without considering the influence of groundwater.

The simulation of staged construction steps is based on actual construction procedures, outlined as follows:

Condition 1: Completion of dewatering processes, installation of drainage and row of piles;

Condition 2: Excavation to support the crown beam bottom elevation −1.8 m, with the addition of the first support system;

Condition 3: Sequential excavation of soil layers to the bottom elevation of −6.7 m of the second support system, followed by the installation of wale and the second tier of support;

Condition 4: Layered excavation of soil down to the slab bottom at an elevation of −11.25 m.

### 4.2. Numerical Simulation Results

To validate the feasibility of selected support structures and the accuracy of numerical simulation results, initial input parameters were employed to calculate representative displacement values at monitoring points PD21 (vertical displacement of pile top) and CX07 (horizontal displacement along the pile shaft).

(1) Vertical displacement of pile top

**Figure 8:**
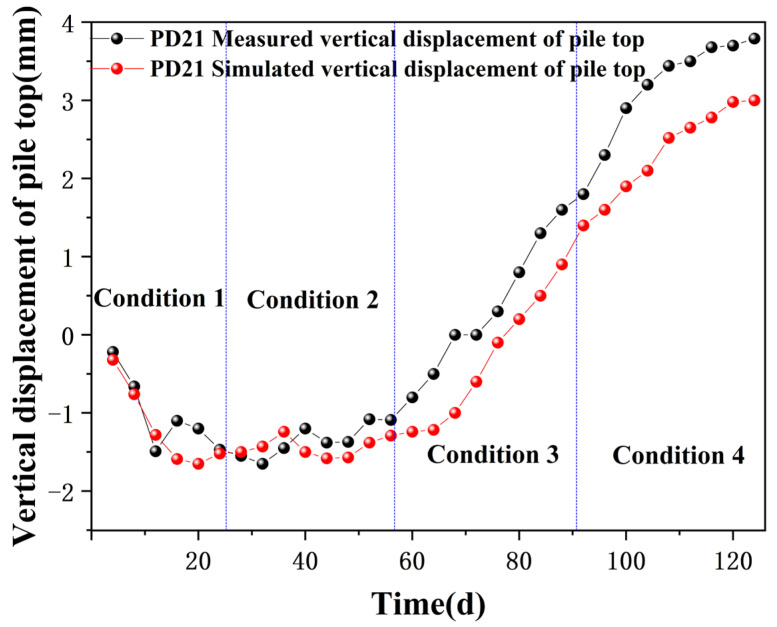
Vertical Displacement at PD21.

Figure 8 reveals that during the excavation and supporting phases, the vertical displacement at PD21 fluctuates, mirroring the trend observed in field monitoring data, albeit with larger amplitude fluctuations. The continued increase in displacement at the pile top during the later stages of construction is attributed to the excavation process exposing the pit bottom, causing a slight upward shift of the surrounding piles due to heaving at the pit base. In later construction phases, the field-measured values exceed the simulation results of the pile top displacement. This discrepancy arises because finite element software cannot precisely mimic the excavation process stage by stage, and the simulated phases are often completed earlier than the actual excavation, leading to differences between measured and simulated outcomes.

**Figure 9:**
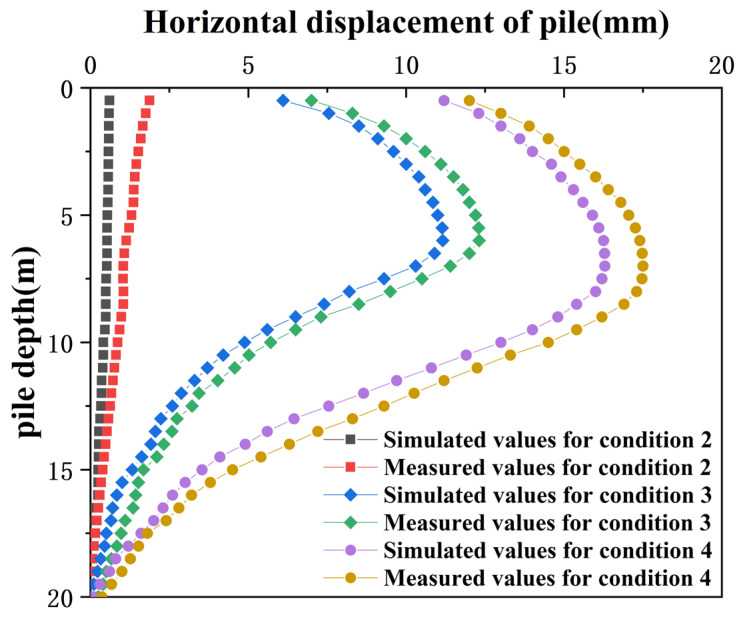
Horizontal displacement along the pile shaft.

Pit excavation to the bottom of the pit, under different working conditions CX07 horizontal displacement of pile simulated values and measured values as shown in Figure 9. When the foundation pit is excavated to the bottom elevation, the simulated and measured values of the horizontal displacement of pile CX07 are presented in Figure 9. A horizontal displacement is taken every 0.5 m from the top of pile CX07 to the bottom of the pile; Figure 9 shows the monitoring value at the end of construction. Field measurements indicate that internal bracing effectively mitigates horizontal displacement along the pile shaft, affirming the adequacy of the support and bracing design. During the excavation to a shallow depth in condition two, the horizontal displacement decreases progressively from the pile top to the bottom. In conditions three and four, the horizontal displacement of the pile increases first and then decreases from top to bottom; as the excavation depth increases, the position of maximum horizontal displacement moves downward. The maximum measured horizontal displacement was 17.51 mm, compared to the simulated maximum of 16.29 mm. Both measured and simulated displacements remained within the warning thresholds, suggesting that the numerical model accurately reflects changes in horizontal displacement along the pile shaft.

Overall, although there are uncontrollable discrepancies between the numerical results and field measurements, the trends are generally consistent. This demonstrates that the use of PLAXIS 3D for simulating deep excavation and deformation effectively captures the dynamic changes in the displacement of support structures, and numerical simulation can reliably predict pit deformations and accurately reflect the stability and safety of the excavation.

### 4.3. Impact of Parameters on Simulation Outcomes

The use of the HSS model for numerical simulation indicates that it is challenging to ascertain soil stiffness parameters $E_{50}^{ref}$, $E_{ur}^{ref}$, and $E_{oed}^{ref}$, often leading to empirical estimates within a reference range, which introduces deviations between simulation results and actual field conditions. The effective Friction angle ($\varphi^{\prime }$) and effective cohesion ($c^{\prime }$) directly influence the shear strength and stability of the soil, impacting the output of numerical simulations of excavation and support. During the dynamic construction process of a deep pit, the soil within the pit undergoes vertical unloading, leading to dynamic changes in stress states and continuous variations in soil parameter values, affecting the overall stability of the excavation. By evaluating the maximum horizontal displacement at monitoring point CX01, the influence of soil parameters $E_{50}^{ref}$, $E_{ur}^{ref}$, $E_{oed}^{ref}$, $\varphi^{\prime }$, and $c^{\prime }$ on the numerical simulation results is determined by the method of controlling variables. By changing the values of the original soil parameters to 0.7 and 1.3 times the original, and other parameter values remain unchanged, the numerical simulation calculation results are shown in Table 2.

Table 2 indicates that the impact of soil parameters on the results of numerical simulations varies in the order of $\varphi^{\prime }$>$E_{50}^{ref}$>$E_{oed}^{ref}\approx c^{\prime }$>$E_{ur}^{ref}$, with parameters $E_{oed}^{ref}$ and $c^{\prime }$ having a similar effect. Since parameter $c^{\prime }$ can be determined through in-lab geotechnical testing, this study focuses on the inversion of soil parameters $\varphi^{\prime }$, $E_{50}^{ref}$, and $E_{oed}^{ref}$, which significantly affect the outcomes.

Parameter inversion was performed for soil layers spanning from the top to the bottom of the pit, encompassing five distinct layers. However, due to the negligible displacement changes observed during the excavation of the first layer, this study only conducts parameter inversion for the second through fifth layers. This approach allows the input parameters in PLAXIS 3D to more closely align with the actual soil parameters encountered during the construction process, enhancing the accuracy and relevance of the numerical model.

## 5. Dynamic Prediction Analysis of Deep Pit

### 5.1. Sample Collection

Parameters to be inverted are classified as in Table 3, and the prefixed numbers indicate the i-th soil layer. The setup involves 12 parameters—take three levels for each factor. Utilizing an orthogonal array of twelve factors at three levels (Table $L_{27}(3^{12})$), 27 datasets were constructed to serve as input parameters for PLAXIS 3D. The horizontal displacement of the CX07 pile shaft at intervals of 0.5 m was selected as the output for PLAXIS 3D. Each dataset produced a corresponding set of output values, creating a total of 27 datasets that were employed as training samples for the PSO-BP neural network.

### 5.2. Results of PSO-BP Parameter Inversion

**Figure 10:**
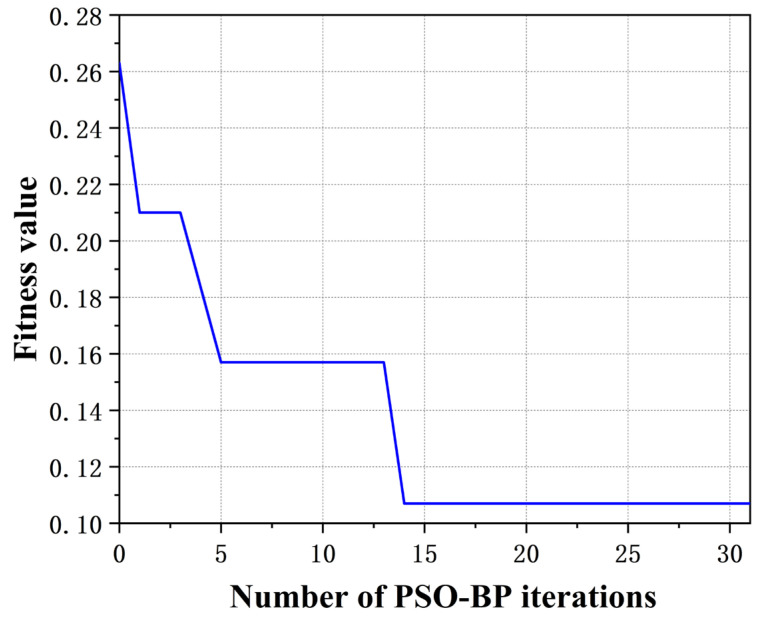
Convergence Speed of the PSO-BP Iterations.

The PSO-BP was developed using the MATLAB toolbox. The architecture of the neural network was configured with 40 nodes in the input layer, 12 nodes in the output layer, and an optimal number of 10 nodes in the hidden layer. Leveraging the global optimization capabilities of the PSO algorithm optimized weights and thresholds were assigned to the network, enhancing the accuracy and efficiency of the model. The convergence speed of the PSO-BP iterations is illustrated in Figure 10.

Figure 10 shows that the PSO-BP achieves the best fitness value in the 14th generation. The results of the program indicate that the PSO can enhance the convergence speed and optimization performance of the BP neural network, thus improving its tendency to become stuck in local optima.

To achieve dynamic prediction throughout the excavation process of the pit, the field-measured data of pile CX07 after the completion of excavation under condition 2 is used as the input sample for the PSO-BP neural network. The output is the soil parameter values under condition 2, which are then used as input parameters for PLAXIS 3D to predict the displacements under condition 3. After the actual excavation is completed, the measured data are used as an input sample in the same manner to inversely calculate the soil parameter values under condition 3. These updated parameters are then used to predict the displacements under condition 4 in PLAXIS 3D. The inversely calculated parameter values are shown in Table 4.

### 5.3. Dynamic Prediction Results Analysis

**Figure 11:**
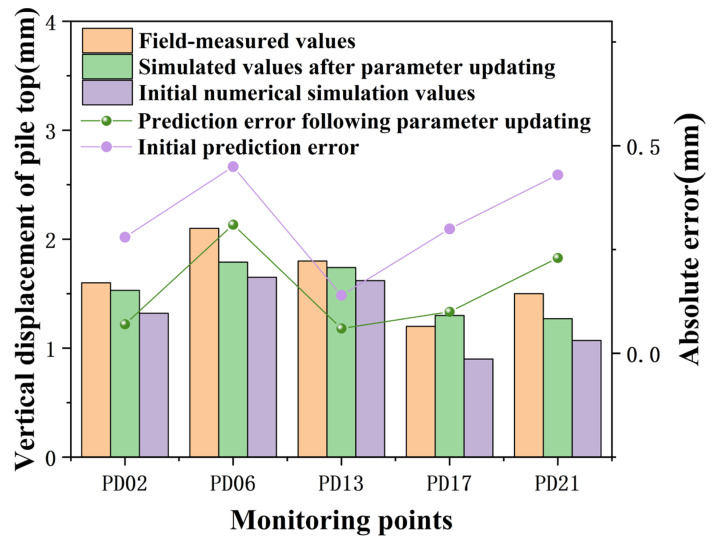
Vertical displacement at the top of pile PD07 during condition 3.

To minimize errors in the computational results of PLAXIS 3D, parameters inverted from measured data during condition 2 were used as inputs. This enabled the calculation of deformations in the support structure for condition 3, with a comparative analysis against typical in-pit pile measurements. Following the excavation completion, comparisons between measured and simulated vertical displacements at the pile top for condition 3 are illustrated in Figure 11, and comparisons of maximum horizontal displacements along the pile body are presented in Table 5.

Figure 11 shows that during condition 3, the maximum vertical displacement at the pile top across five measurement points was 2.1 mm, with a minimum of 1.2 mm. The absolute errors between the initial numerical simulation results and the actual measurements were greater than those between the updated parameters’ predictions and the actual measurements, with an average absolute error of 0.4 mm using initial parameters and 0.19 mm after parameter updating.

Table 5 indicates that after the inversion of soil parameters using PSO-BP based on measured displacements from condition 2, the simulation results for maximum horizontal displacement along of body align closely with actual measurements. Comparing both prediction results and field measurements, the average absolute error for horizontal displacements calculated using initial parameters was 1.41 mm, which was reduced to 0.93 mm after updating the parameters.

**Figure 12:**
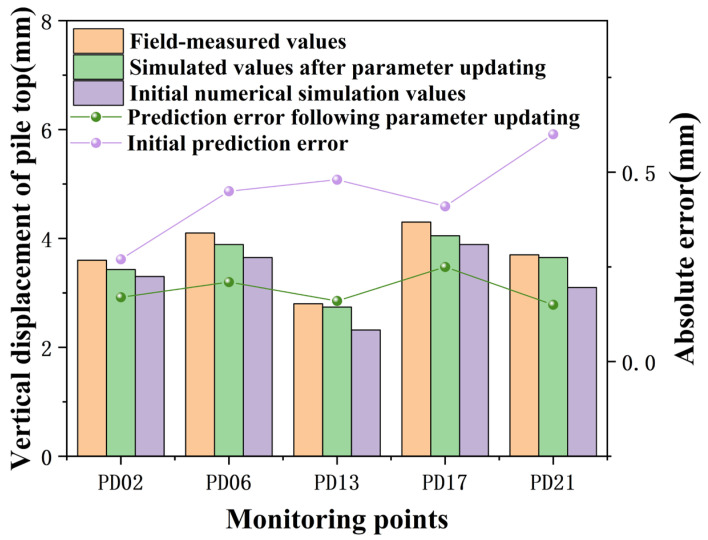
Vertical displacement at the top of pile PD07 during condition 4.

Upon entering the measured displacement values from condition 3 into the trained neural network model, the output soil parameters were used to simulate excavation and support processes for condition 4 in PLAXIS 3D. Comparisons of measured and simulated vertical displacements at the pile top for condition 4 are depicted in Figure 12, and comparisons of maximum horizontal displacements along the pile body are shown in Table 6.

Figure 12 illustrates that during condition 4, the maximum vertical displacement at the pile top across five measurement points was 4.3 mm, with a minimum of 2.8 mm. Using the initial numerical model, the maximum absolute error between field measurements and simulation results was 0.48 mm, which decreased to 0.25 mm after parameter updating. The average absolute error for vertical displacements at the pile top calculated using initial parameters was 0.44 mm, which was reduced to 0.19 mm after updating the parameters, demonstrating that the updated parameter values more closely reflect the actual conditions of the project and updating the input parameters in PLAXIS 3D can decrease the absolute error between predicted and measured values of pile top deformations.

Table 6 reveals that the absolute errors in model calculation results after parameter updating were smaller than those of the initial model. During condition 4, the maximum absolute error for horizontal displacements along of pile body at four measurement points ranged from 0.93 mm to 0.25 mm, with an average of 0.59 mm, all lower than the errors between initial simulation results and actual measurements.

The comprehensive analysis indicates that numerical simulation results after parameter updating closely match actual measurements, with absolute errors consistently lower than those of the initial simulation results. This confirms the feasibility of dynamically predicting the excavation process by inverting soil parameters based on field measurements from preceding conditions. While the study assumed a uniform distribution of soil layers in PLAXIS 3D modeling and selected parameters that significantly impacted the results, a sensitivity analysis on different soil layer parameters was not conducted, marking a limitation of this research. Nonetheless, this parameter inversion method has been effectively validated by an actual engineering project, significantly reducing the absolute errors between numerical simulations and field measurements, offering insights for similar projects and guiding soil parameter selection in the region.

## 6. Conclusions

Through the establishment of a numerical model using PLAXIS 3D and inversion of soil parameters using the PSO-BP neural network to update input parameters of the model, this study has successfully implemented dynamic predictions throughout the entire process of deep excavation. The feasibility of the inverted parameters was verified with actual project data, confirming the effectiveness of the model updates.

Specific conclusions are as follows:

(1) Utilizing the Particle Swarm Optimization to enhance the BP neural network for soil layer parameter inversion significantly improves the convergence speed of the model and prevents the common issue of the network becoming trapped in local optima;

(2) The actual changes in vertical displacements at the pile top and horizontal displacements along the pile body during the excavation and support process are generally consistent with those predicted by the simulation; the absolute errors between initial numerical simulation results and measured values are greater than those between updated predicted values and measured values;

(3) Dynamic prediction results are closer to actual measurements compared to initial numerical simulations. The methodology of inverting soil layer parameters based on field measurements from previous conditions is feasible and provides valuable insights for selecting soil parameters in the region.

While this study has achieved dynamic prediction throughout the entire process of pit excavation, there are still limitations, specifically manifesting in the following aspects:

(1) The numerical simulation adopted in this paper for the excavation and deformation monitoring of pits did not take into account the dynamic loads around the pit perimeter, as well as the influence of existing surrounding structures on the retaining system. Due to the reduction of the groundwater level to the bottom elevation of the pit before construction, the soil humidity and suction state above the groundwater level were not considered in the numerical simulation;

(2) In the future, the authors will focus on investigating the influence of soil parameters on the displacement of the retaining structure of pits, as well as the impact of multiple soil parameters from different soil layers in the excavation area on the results of numerical simulations.

## Figures and Tables

**Table 1:** Basic Parameters of Pit Soil Layers.

Soil Layer	Thickness (m)	Weight Density (KN/m^3^)	Friction Angle (°)	Cohesion (KPa)	Compression Modulus (MPa)
1-1 plain fill	2.4	18.3	10.0	12.0	5.34
1-2 plain fill	2.2	18.4	8.0	15.0	6.38
3 clay	1.6	19.5	14.3	52.7	7.43
4-1 silty clay	2.1	18.7	14.3	27.6	5.94
4-2 silt with silt-sand	4.1	18.6	24.5	8.0	10.20

**Table 2:** Impact of Soil Parameters on Numerical Simulation Outcomes.

Changing Factors	/	$\varphi^{\prime }$ (°)		$c^{\prime }$ (Kpa)		$E_{50}^{ref}$ (MPa)		$E_{ur}^{ref}$ (Mpa)		$E_{oed}^{ref}$ (Mpa)	
Multiple of change	1.0	0.7	1.3	0.7	1.3	0.7	1.3	0.7	1.3	0.7	1.3
Maximum value (mm)	18.56	19.34	18.15	18.62	18.34	18.04	19.18	18.28	18.69	18.44	18.74
Rate of change (%)	/	4.20	2.21	0.32	1.19	2.81	3.34	1.51	0.70	0.65	0.97

**Table 3:** Soil Layer Parameter Level Classification.

	$2E_{50}^{ref}$	$2E_{ur}^{ref}$	$2\varphi^{\prime }$	$3E_{50}^{ref}$	$3E_{ur}^{ref}$	$3\varphi^{\prime }$	$4E_{50}^{ref}$	$4E_{ur}^{ref}$	$4\varphi^{\prime }$	$5E_{50}^{ref}$	$5E_{ur}^{ref}$	$5\varphi^{\prime }$
Unit	MPa	MPa	°	MPa	MPa	°	MPa	MPa	°	MPa	MPa	°
Level 1	5.38	16.14	4.80	6.43	19.29	8.58	4.94	14.82	8.58	9.20	27.60	14.70
Level 2	6.38	25.52	8.00	7.43	19.72	14.3	5.94	23.76	14.30	10.20	40.80	24.50
Level 3	7.38	36.90	11.20	8.43	42.15	20.02	6.94	34.70	20.02	11.20	56.00	34.30

**Table 4:** Parameter values inverted under different conditions.

	$2E_{50}^{ref}$	$2E_{ur}^{ref}$	$2\varphi^{\prime }$	$3E_{50}^{ref}$	$3E_{ur}^{ref}$	$3\varphi^{\prime }$	$4E_{50}^{ref}$	$4E_{ur}^{ref}$	$4\varphi^{\prime }$	$5E_{50}^{ref}$	$5E_{ur}^{ref}$	$5\varphi^{\prime }$
Unit	MPa	MPa	°	MPa	MPa	°	MPa	MPa	°	MPa	MPa	°
Starting value	6.38	25.52	8.00	7.43	19.72	14.3	5.94	23.76	14.30	10.20	40.80	24.50
Condition 2	6.42	26.06	7.80	7.86	24.19	11.44	6.28	28.17	12.88	11.55	46.12	22.14
Condition 3	6.55	28.46	7.15	7.97	28.38	13.58	6.54	26.43	14.19	13.74	51.51	20.75

**Table 5:** Comparison of maximum horizontal displacement of pile body.

Monitoring Point	Measured Displacement/mm	Numerical Simulation Results		Predicted Value after Parameter Update	
		Calculation Result (mm)	Absolute Error (mm)	Calculation Result (mm)	Absolute Error (mm)
CX01	12.02	10.68	1.34	11.84	0.18
CX03	13.38	11.99	1.39	13.07	0.31
CX07	13.57	12.24	1.33	13.38	0.19
CX11	13.74	12.16	1.58	13.49	0.25

**Table 6:** Comparison of maximum horizontal displacement of pile body.

Monitoring Point	Measured Displacement/mm	Numerical Simulation Results		Predicted Value after Parameter Update	
		Calculation Result /mm	Absolute Error /mm	Calculation Result/mm	Absolute Error/mm
CX01	18.56	17.54	1.02	17.63	0.93
CX03	17.64	16.01	1.63	17.05	0.59
CX07	17.51	16.29	1.22	16.94	0.57
CX11	21.96	20.58	1.38	21.71	0.25

## Data Availability

Data are contained within the article.
